# 'Is tinnitus accompanied by hemifacial spasm in normal-hearing patients also a type of hyperactive neurovascular compression syndrome? : A magnetoencephalography study

**DOI:** 10.1186/1471-2377-13-42

**Published:** 2013-05-08

**Authors:** Won Seok Chang, Bong Soo Kim, Ji Eun Lee, Hyun Ho Jung, Kiwoong Kim, Hyuk Chan Kwon, Yong Ho Lee, Jin Woo Chang

**Affiliations:** 1 Department of Neurosurgery, Brain Research Institute, Yonsei University College of Medicine, Seoul, Korea; 2 Korea Research Institute of Standard and Science (KRISS), Daejeon, Korea; 3 Department of Neurosurgery, Brain Research Institute, Yonsei University College of Medicine, 134 Shinchon-Dong, Seodaemoon-Gu, Seoul, 120-752, Korea

**Keywords:** Hemifacial spasm, Magnetoencephalography, Pathophysiology, Tinnitus

## Abstract

**Background:**

Traditionally, tinnitus accompanied by hemifacial spasm has been considered a type of hyperactive neurovascular compression syndrome that is similar to hemifacial spasm alone because of the anatomically close relationship between the facial nerve and cochlear nerve as well as the hyperactive clinical nature.

**Methods:**

Participants were 29 subjects who presented with hemifacial spasm and neuroradiological evidence of vascular compression of the cranial (facial/cochlear) nerve. We used magnetoencephalography (MEG) to estimate the activity of the cochlear nerve in patients with and without tinnitus on the ipsilateral side. We compared the difference in the latency and the ratio of the equivalent current dipole (ECD) strength between the ipsilateral and contralateral sides of the spasm and tinnitus.

**Results:**

Cochlear nerve activity in patients with tinnitus was increased with a shorter latency (p = 0.016) and stronger ECD strength (p = 0.028) compared with patients without tinnitus.

**Conclusion:**

The MEG results from normal-hearing patients who had tinnitus accompanied by hemifacial spasm suggest that the hyperactivity of the auditory central nervous system may be a crucial pathophysiological factor in the generation of tinnitus in these patients. The neurovascular compression that causes sensory input from the pathologic facial nerve activity may contribute to this hyperactivity of the central auditory nervous system.

## Background

Tinnitus is defined as the subjective perception of a sound in the absence of any physical sound source. Tinnitus may be the result of spontaneous and aberrant neural activity of the auditory system. Approximately 5-15% of the population in western societies experience chronic tinnitus for more than 6 and up to 12 months [1]. A wide range of therapies including pharmacological and surgical intervention have been proposed for the treatment of tinnitus symptom. Because tinnitus usually coincides with various ear disorders, surgical treatment of chronic tinnitus mainly focuses on ontological surgery. However, some types of tinnitus such as pulsatile tinnitus, which can be caused by vascular compression of the auditory nerve, may require different therapeutic approaches.

Microvascular decompression surgery has been used to treat tinnitus because neurovascular compression of the cochlear nerve is assumed to be one of the causes of tinnitus. And this type of Tinnitus is similar to other neurovascular compression syndromes, such as hemifacial spasm (HFS) and trigeminal neuralgia [2-5]. Interestingly, some patients experience ipsilateral tinnitus accompanied by HFS. This type of tinnitus was encountered in 10 of 142 patients with HFS in a study by Ryu et al. [6]. If tinnitus is accompanied by HFS, the surgical outcome following microvascular decompression has a relatively high success rate, especially in cases in which the cochlear nerve is affected [6,7]. It has been suggested that some forms of tinnitus may be caused by neurovascular compression in the cerebellopontine angle [8,9].

The pathophysiology of HFS has been relatively well studied [10]. However, the pathophysiology of tinnitus is still controversial, especially if tinnitus is accompanied by HFS. To investigate the pathophysiology of tinnitus accompanied by HFS, we used magnetoencephalography (MEG) to study patients and analyzed the relationship between the presence of tinnitus and the MEG results.

## Methods

### Participants

Participants were 29 subjects with HFS with neuro-radiological evidence of vascular compression of cranial (facial/auditory) nerve. Inclusion criteria were as follows: 1) patients with unilateral HFS; 2) patients with tinnitus on the same side as the HFS, if the patient had tinnitus; 3) patients with a hearing level better than 20 dB at 1000 Hz and better than 25 dB at each frequency examined (250 Hz to 3000 Hz with pure tone audiometry, as measured by an otorhinolaryngologist); 4) patients without otologic disorders; 5) patients with differences in hearing levels between the left and right ear of 5 dB or less; and 6) patients who could undergo MEG and magnetic resonance imaging (MRI) procedures. The diagnosis of HFS was made according to clinical symptoms and MRI findings of vascular compression of the cranial (facial/cochlear) nerve. From January 2011 to December 2011, 29 patients met these criteria and underwent MEG.

This study was approved by the Korean Food and Drug Administration. All participants provided written informed consent, and the study was approved by the Institutional Review Board of Severance Hospital, Seoul, Korea (IRB no. 1-2011-0088).

### Stimulation and MEG measurement

Tone bursts of 100-ms duration (10-ms slope) were employed for the acoustic stimulation protocol. Pure tones of 1000 Hz were applied to the patient’s left and right ear. The acoustic stimulation consisted of 100 epochs of a random inter-stimulus interval between 900 and 1000 ms. Individual hearing thresholds were determined before the stimulation. Tones were delivered at a comfortable level 40 dB above threshold through two 2.5-m long silicon tubes (ER-30, Etymotic Research, Inc., USA). Sound stimuli were generated with a STIM2 system (Compumedics Neuroscan, USA).

MEG data for all patients were recorded with a whole-head MEG system (KRISS, Daejeon, Korea) with 152 axial first-order gradiometers. Sound-triggered epochs (including a 100-ms pre-stimulus baseline) were filtered online with a bandpass of 0.1-100 Hz and recorded at a sampling rate of 1000 Hz.

The measured auditory evoked field (AEF) waveforms were filtered offline with 3–40 Hz band-pass filtering, and the neuromagnetic responses to the auditory stimuli were averaged to improve the signal-to-noise ratio. The baseline for the waveforms was defined as a mean amplitude between −100 and 0 ms relative to the tone onset.

We selected neuromagnetic data in the hemisphere contralateral to the stimulus to calculate equivalent current dipoles (ECDs) [5]. The dipolar moment at the N100m response was used to detect the peak latency of the N100m and to explain the magnetic field patterns during the N100m (70–140 ms). The goodness-of-fit (%) of the N100m dipole was calculated and merged with the patient’s MRI to verify the anatomical location of the N100m dipole. To compare the effect of each clinical factor on the N100m latency and ECD strength, the latency difference of the N100m (the latency of the N100m on the tinnitus side − the latency of the N100m on the control side) and the ratio of the ECD strength of the N100m (the ECD strength of the N100m on the tinnitus side/the ECD strength of the N100m on the control side) were calculated.

Clinical data and MEG data were analyzed together. To compare the N100m latency and ECD strength, the latency difference of the N100m and the ratio of ECD strength of the N100m were analyzed by paired *t*-test and non-parametric Mann–Whitney tests. All statistical tests were performed using SPSS v.18.0 (NCSS statistical software, Kaysville, UT, USA). All statistical tests were two-tailed. The threshold for statistical significance was set at p < 0.05.

## Results

**Figure 1:**
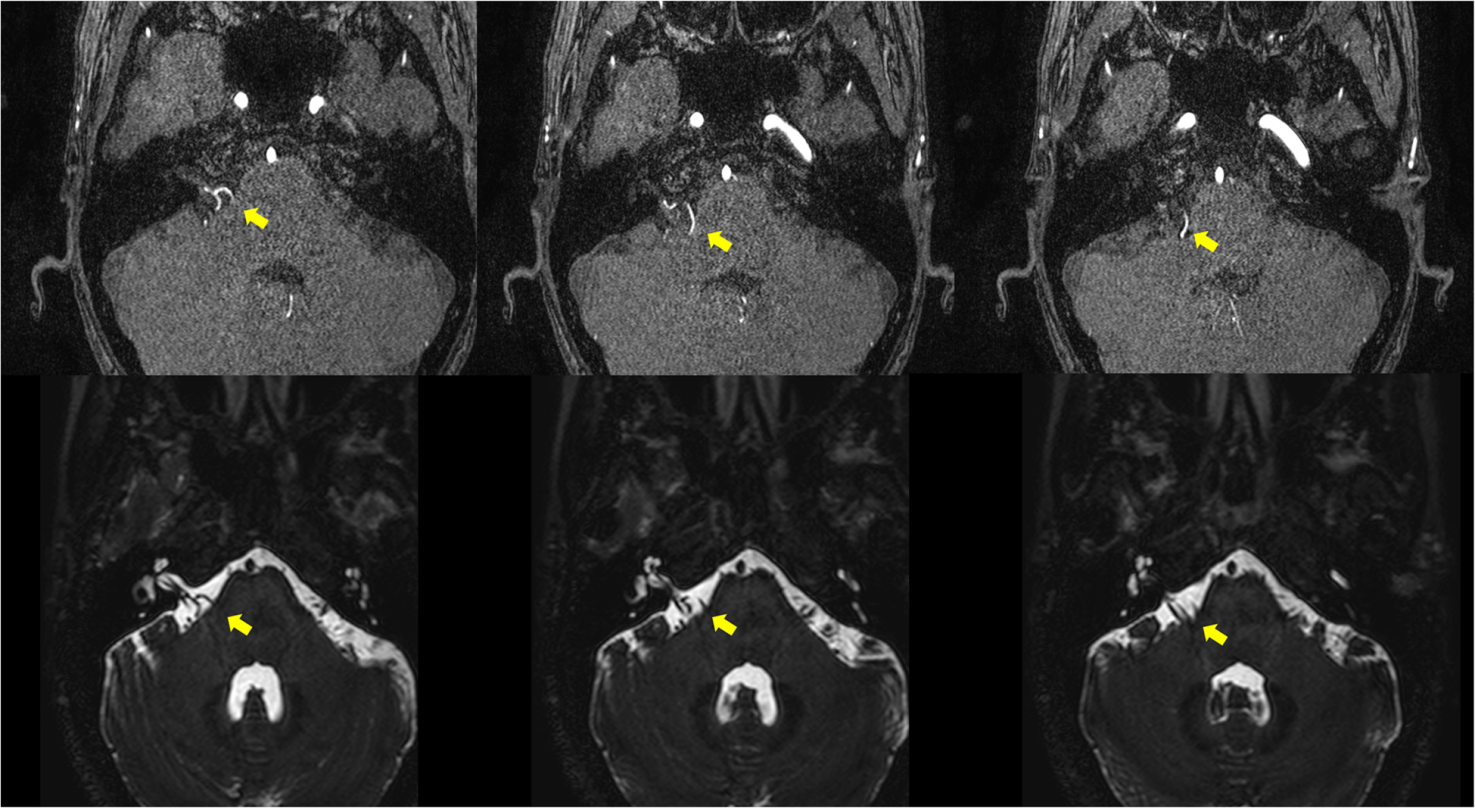
**A magnetic resonance image of a patient with tinnitus accompanied by HFS.** Upper row: The source image from a three-dimensional time-of-flight image. Lower row: A three-dimensional Fourier transformation with constructive interference in the steady-state image. Arrow: The site of neurovascular compression of the facial and vestibulocochlear nerve complex by a branch from the posterior inferior cerebellar artery.

The 29 patients included 6 males and 23 females with a mean age of 48.9 years (range, 33–69 years). The most frequent offending vessels were the anterior inferior cerebellar artery, followed by the posterior inferior cerebellar artery (Figure 1). Among all patients, only eight patients had ipsilateral tinnitus, which was low-pitch pulsatile in six patients and high-pitch continuous in two patients (Table 1). After microvascular decompression, the HFS of all of the patients with tinnitus was completely resolved. Furthermore, the tinnitus of seven of the eight patients with tinnitus disappeared after surgery. One patient with high-pitch continuous tinnitus continued to suffer from tinnitus after surgery.

**Table 1:** Patient demographic characteristics

**Total**				**29**
	Gender	Male	6	
		Female	23	
	Age (years)	Mean (Range)	48.9 (33–69)	
	Offending vessel	AICA	15	
		PICA	14	
	Tinnitus	Yes	8	
		No	21	
	Type of tinnitus	Low-pitch pulsatile	6	
		High-pitch continuous	2	

Abbreviations: AICA, anterior inferior cerebellar artery; PICA, posterior inferior cerebellar artery.

**Figure 2:**
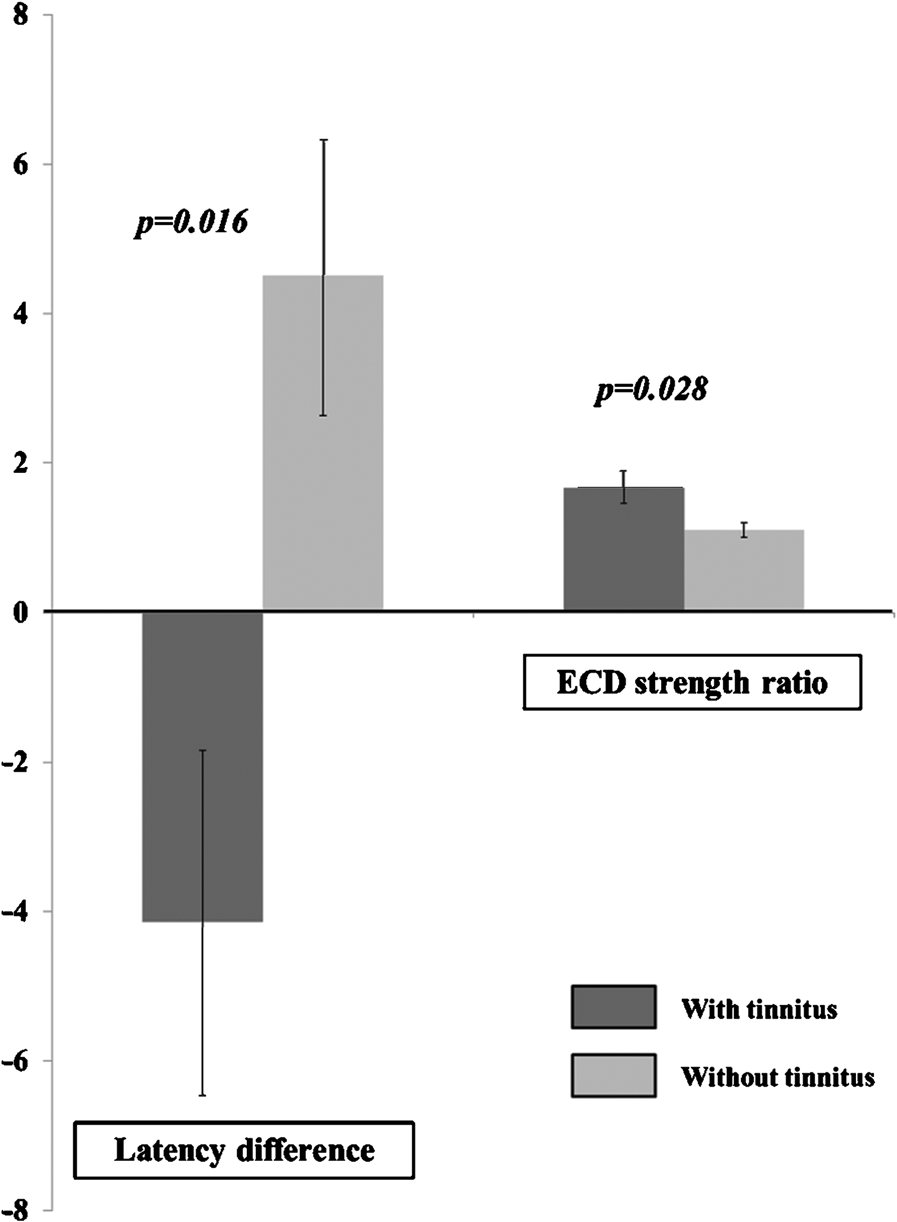
A comparison of the difference in the N100m latency and the ratio of equivalent current dipole strength (ECDs) of N100m between the control side and HFS side.

**Figure 3:**
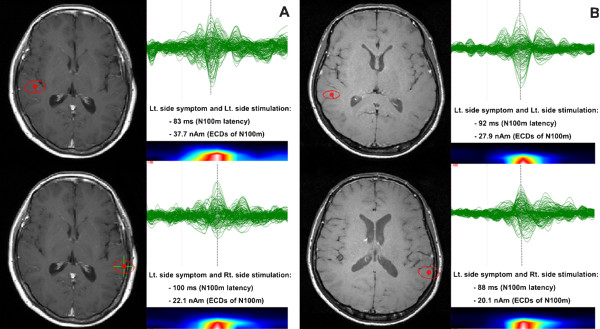
**An example of the average dipoles after auditory stimulation (A and B).** The ECDs of the N100m, and the N100m latency of a patient with tinnitus accompanied by left-side HFS are greater and faster after auditory stimulation on the HFS side (**A**). However, these findings were not observed in patients without tinnitus (**B**).

There were no statistical differences in the N100m latency and ECD strengths between the results of the AEF waveform on the control side and the AEF waveform on the HFS side across all patients. The mean N100m latencies of the control and HFS sides across all patients were 99.1 ± 10.0 ms (mean ± s.d.) and 101.3 ± 14.3 ms, respectively (paired *t*-test, p = 0.198) (Table 2). The mean ECD strengths of the control and HFS sides across all patients were 24.8 ± 11.6 nAm and 27.5 ± 9.4 nAm, respectively (paired *t*-test, p = 0.265) (Table 3). However, the latency difference in the N100m between the control side and the HFS side was smaller in patients with tinnitus compared with patients without tinnitus (−4.1 ± 6.5 with tinnitus vs. 4.6 ± 8.6 without tinnitus, Mann–Whitney test, p = 0.016). One interpretation of this result is that the N100m latencies on the HFS side in patients with tinnitus were much shorter than the N100m latencies on the control side. This result was not evident in patients without tinnitus (Figures 2 and 3). The ratio of the ECD strength was greater in patients with tinnitus compared with patients without tinnitus (1.7 ± 0.6 with tinnitus vs. 1.1 ± 0.4 without tinnitus, Mann–Whitney test, p = 0.028) (Figures 1 and 3). However, the latency difference of the N100m and the ratio of the ECD strength of the N100m were not significantly different according to gender, age, or offending vessel.

**Table 2:** Summary of results (N100m latency)

**Total**	**Clinical factors**		**Auditory stimulation side**	**N100m latency**	***p *value**
				**(ms, mean ± S.D.)**	
Total			HFS side	101.3 ± 14.3	0.198^†^
			Control side	99.1 ± 10.0	
	Tinnitus	yes	HFS side	92.8 ± 7.9	**0.016**^**‡**^
			Control side	97.0 ± 7.1	
		no	HFS side	104.5 ± 15.0	
			Control side	100.0 ± 10.9	
	Gender	Male	HFS side	98.5 ± 9.0	0.546^**‡**^
			Control side	98.3 ± 7.4	
		Female	HFS side	102.0 ± 15.5	
			Control side	99.1 ± 10.0	
	Age (years)	≥50	HFS side	104.5 ± 15.1	0.949^**‡**^
			Control side	100.0 ± 10.9	
		<50	HFS side	98.9 ± 7.9	
			Control side	97.0 ± 7.1	
	Offender	AICA	HFS side	95.7 ± 9.0	0.333^**‡**^
			Control side	97.3 ± 7.8	
		PICA	HFS side	107.3 ± 16.7	
			Control side	101.0 ± 11.8	

^†^ N100m latency of HFS side vs. N100m latency of control side, paired *t*-test.

^‡^ Comparison of N100m difference according to each factor, Mann–Whitney test.

* Latency of N100m on the HFS side − latency of N100m on the control side.

Abbreviations: AICA, anterior inferior cerebellar artery; PICA, posterior inferior cerebellar artery; S.D., standard deviation.

**Table 3:** Summary of results (ECD strength)

**Total**	**Clinical factors**		**Auditory stimulation side**	**ECD strength**	***p *value**
				**(nAm, mean ± S.D.)**	
Total			HFS side	27.5 ± 9.4	0.265^†^
			Control side	24.8 ± 11.6	
	Tinnitus	yes	HFS side	30.5 ± 12.7	**0.028**^‡^
			Control side	18.8 ± 5.1	
		no	HFS side	26.4 ± 8.0	
			Control side	27.1 ± 12.6	
	Gender	Male	HFS side	27.6 ± 9.3	0.694^‡^
			Control side	25.6 ± 10.4	
		Female	HFS side	27.5 ± 10.2	
			Control side	24.6 ± 12.1	
	Age (years)	≥50	HFS side	30.31 ± 1.0	0.234^‡^
			Control side	29.3 ± 12.4	
		<50	HFS side	24.6 ± 6.5	
			Control side	20.0 ± 9.6	
	Offender	AICA	HFS side	26.5 ± 10.5	0.201^‡^
			Control side	22.0 ± 12.4	
		PICA	HFS side	28.7 ± 8.4	
			Control side	27.9 ± 10.2	

^†^ ECD strength of HFS side vs. ECD strength of control side, paired *t*-test.

^‡^ Comparison of the ratio of ECD strength according to each factor, Mann–Whitney test.

* ECD strength of N100m on the HFS side/ECD strength of N100m on the control side.

Abbreviations: AICA, anterior inferior cerebellar artery; ECD, equivalent current dipole; PICA, posterior inferior cerebellar artery; S.D., standard deviation.

## Discussion

The concept of a hyperactive neurovascular compression syndrome such as HFS and trigeminal neuralgia has been accepted since Dandy’s first report, and microvascular decompression for these syndromes has acceptable surgical outcomes [11-14]. Due to the anatomically close relationship between the cochlear nerve and the facial nerve, some attempts have been made to perform microvascular decompression to treat tinnitus, based on the assumption that tinnitus is a type of hyperactive neurovascular compression syndrome. However, the response rate of microvascular decompression for tinnitus varies from 40 to 77%, and the tinnitus-free rate is lower than the response rate [2-5]. In contrast, an interesting report about tinnitus accompanied by HFS has been published. Ryu et al. investigated the results of microvascular decompression for tinnitus accompanied by HFS. Tinnitus in 7 of 10 (70%) patients was completely resolved after surgery, which is comparable to the surgical result for HFS and trigeminal neuralgia [6]. This unexpectedly high success rate may be related to the differences in clinical characteristics of patients with HFS in whom offending vessels run very close to the cochlear nerve. However, neurovascular compression has been evident on MRI in tinnitus patients without HFS in other reports. Therefore, the pathophysiologic mechanism of tinnitus accompanied by HFS may be different from the pathophysiological mechanism of tinnitus alone.

Initially, we assumed that the pathophysiological mechanism of tinnitus accompanied by HFS might be quite similar to the two suggested pathophysiological mechanisms of hyperactive neurovascular compression syndrome, ephaptic transmission and hyperactive cranial nerve nucleus and/or higher brain structure. If tinnitus accompanied by HFS is caused by ephaptic transmission of the cochlear nerve, then MEG for these patients should have shown decreased auditory cortical activity and a delayed latency from the auditory stimulus and cortical evoked field. However, MEG results of the AEF waveform for our patients with tinnitus accompanied by HFS showed simultaneously increased auditory cortical activity and decreased N100m latency on the HFS side compared with patients with HFS without tinnitus (Tables 2 and 3). This result suggests that tinnitus accompanied by HFS is unlikely to be caused by ephaptic transmission of the cochlear nerve by neurovascular compression. Therefore, hyperactivity and hyper-conductivity of the central auditory nervous system may play a key role in the pathophysiological mechanism [10].

A recent study was published about normal-hearing patients with tinnitus. In this study, the authors observed a shortening of the I-V latency and enlarged Na and Pa amplitudes, and concluded that the cause of tinnitus in these patients seemed to have originated from the central nervous system [15]. Although the patients in that study did not have HFS, the tinnitus conditions were similar to our patients. The results of our study suggest that tinnitus may originate in the central nervous system, rather than the cranial nerve or the root entry zone.

Although the pathophysiology of tinnitus is still controversial, an interesting theory regarding the pathophysiological mechanism of tinnitus has been introduced recently. According to the theory, the dorsal cochlear nucleus in the pons, which is modulated by multi-sensory input, is a strong candidate for the origin of tinnitus [16-18]. Multi-sensory input can affect dorsal cochlear nucleus granule cells, leading to changes in dorsal cochlear nucleus principal cells [19]. Thus, sensory stimuli can modulate cochlear function and may be the cause of tinnitus. Further support for the relationship between sensory stimuli and tinnitus is found in patients with tinnitus who are treated with botulinum toxin. Abnormal movement in the head and neck area can be associated with tinnitus, and this type of tinnitus is successfully cured with botulinum toxin injections into the affected muscle [20,21].

According to our MEG results and recent theories, hyperactivity of the dorsal cochlear nucleus may be one of the major pathophysiological mechanisms of tinnitus accompanied by HFS. In our series, one of eight tinnitus patients, who had high-pitch continuous tinnitus accompanied by HFS, continued to suffer from tinnitus even though there was no evidence of facial spasm after microvascular decompression. These results are comparable with previous outcome reports in which 2 out of 10 patients continued to experience tinnitus after successful microvascular decompression for HFS, although some of these patients experienced a decrease in hearing ability prior to surgery [6]. An irreversible change in the central auditory nervous system may be attributable to the symptoms of these patients, and further study might be helpful in understanding this change.

Hyperactivity of the central auditory nervous system should also be considered in the pathophysiological mechanism of tinnitus accompanied by HFS. Hyperactivity of the dorsal cochlear nucleus may be induced by both neurovascular compression of the cochlear nerve and multi-sensory input from facial sensory stimuli. In our study, we could not conclude which factor had more influence on this hyperactivity. A further clinical trial, which involves controlling facial sensory input with botulinum toxic injections instead of microvascular decompression, will be used to determine the cause of central auditory hyperactivity.

## Conclusions

The pathophysiologic mechanism of tinnitus accompanied by HFS in normal-hearing patients is still controversial. From our MEG data, we conclude that the origin of tinnitus in patients with HFS may not be the cranial nerve but the central auditory nervous system. Further investigation and clinical correlation are required to obtain more information.

## Abbreviations

AEF: Auditory evoked field; ECD: Equivalent current dipole; HFS: Hemifacial spasm; MEG: Magnetoencephalography; MRI: Magnetic resonance imaging.

## Competing interests

The authors declare that they have no competing interests.

## Authors’ contributions

WSC organized the research project and wrote the first draft of the manuscript; BSK performed the research project and reviewed the manuscript; HHJ organized the research project and reviewed the manuscript; JEL reviewed and critiqued the revised manuscript; KWK, HCK, and YHL conceived and organized the research project; JWC conceived the research project and reviewed and critiqued the manuscript. All authors read and approved the final manuscript.

## Pre-publication history

The pre-publication history for this paper can be accessed here:

http://www.biomedcentral.com/1471-2377/13/42/prepub
